# Community utilization of a co-created COVID-19 testing program in a US/Mexico border community

**DOI:** 10.1186/s12889-024-20527-4

**Published:** 2024-11-18

**Authors:** Breanna J. Reyes, Stephenie Tinoco Calvillo, Arleth A. Escoto, Angel Lomeli, Maria Linda Burola, Luis Gay, Ariel Cohen, Isabel Villegas, Linda Salgin, Kelli L. Cain, Dylan Pilz, Paul Watson, Bill Oswald, Cesar Arevalo, Jessica Sanchez, Marjorie Richardson, Jennifer Nelson, Pricilla Villanueva, Garrett McGaugh, Ilya Zaslavsky, Robert H. Tukey, Nicole A. Stadnick, Borsika A. Rabin, Louise C. Laurent, Marva Seifert

**Affiliations:** 1 Department of Obstetrics, Gynecology and Reproductive Sciences, University of California, La Jolla, San Diego, CA USA; 2 Herbert Wertheim School of Public Health and Longevity Science, University of California, La Jolla, San Diego, CA USA; 3 Department of Medicine, University of California, La Jolla, San Diego, CA USA; 4 San Ysidro Health, San Ysidro, CA USA; 5 San Diego State University, University of California San Diego Joint Doctoral Program in Public Health, San Diego, CA USA; 6 Department of Psychiatry, University of California, La Jolla, San Diego, CA USA; 7 Altman Clinical and Translational Research Institute Dissemination and Implementation Science Center, University of California, La Jolla, San Diego, CA USA; 8 Child and Adolescent Services Research Center, San Diego, CA USA; 9 County of San Diego Health and Human Services Agency, San Diego, CA USA; 10 The Global Action Research Center, San Diego, CA USA; 11 Laboratory of Environmental Toxicology, Department of Pharmacology, University of California, La Jolla, San Diego, CA United States of America; 12 San Diego Supercomputer Center, University of California, La Jolla, San Diego, CA USA; 13 Kristian Andersen Lab at The Scripps Research Institute, La Jolla, San Diego, CA USA

**Keywords:** COVID-19 testing, COVID-19 positivity, Latino/a, Border community, Co-creation, Culturally responsive

## Abstract

**Background:**

The COVID-19 pandemic exposed several health disparities experienced by underserved and Latino/a communities, including inequitable access to COVID-19 testing.

**Objective and Goals:**

To describe the utilization of a community-driven and culturally-tailored testing model on COVID-19 testing in an underserved Latino/a community in San Diego.

**Methods:**

The Community-driven Optimization of COVID-19 testing to Reach and Engage Underserved Areas for Testing Equity (CO-CREATE) project implemented a community co-designed COVID-19 testing program in partnership with a Federally Qualified Health Center in a US/Mexico border community.

**Results:**

Between May 2021 and March 2023, 24, 422 COVID-19 PCR tests were administered to 13,253 individuals, among whom 93% percent identified as Latino/a, 57% spoke Spanish in the home, and 38% resided in our target community adjacent to the US/Mexico border, San Ysidro. Based on a subset of available county testing data, CO-CREATE accounted for nearly 12% of all COVID-19 tests reported for San Ysidro residents. Over the course of the project, we estimated that nearly 17% of all San Ysidro residents were tested for COVID-19 through the CO-CREATE project.

**Conclusion:**

These findings highlight the success and reach of this culturally responsive and community co-designed COVID-19 testing program, within a Latino/a border community. Future public health interventions should focus on identifying testing barriers and design appropriate strategies to ensure equitable access to resources and testing uptake for all community members.

## Introduction

Latino/a, Black, Indigenous, and people of color (BIPOC) communities experience disproportionately high rates of poor housing conditions and chronic health conditions, which are all associated with increased risks of COVID-19 infection and associated poor health outcomes [1, 2]. These communities also have reduced access to testing resources, which further increases the likelihood of ongoing transmission [2–7]. Consistent with these factors, Latino/a populations have experienced disproportionately higher COVID-19 infection and hospitalization rates compared to non-Latino/a populations and during the peak of the pandemic, Latinos/as were 2.5–8.5 times more likely to be infected with COVID-19, and 2–18 times more likely to die of COVID-19 compared to their non-Latino/a counterparts [2, 8]. These disparities in health outcomes were intensified by lack of insurance coverage and low English proficiency [8–13]. In addition, there was an inequitable distribution of testing sites and access to testing resources by race in the United States (US) a continuation of underrepresentation of minority populations as a result of historical factors (i.e., racism and poverty) [9, 11, 14, 15].

At the start of the pandemic, Latino/a communities adjacent to the US/Mexico border consistently experienced the highest case rates for SARS-CoV-2 within San Diego County [4, 16]. The border community of San Ysidro is host to one of the busiest Ports of Entry between the US and Mexico where individuals lack access to healthcare resources and, during the COVID-19 pandemic, experienced higher possibilities of exposure [4, 5, 17, 18]. In response to these early pandemic statistics, the Community-driven Optimization of COVID-19 testing to Reach and Engage underserved Areas for Testing Equity (CO-CREATE) project was initiated and funded through the National Institutes of Health Rapid Acceleration of Diagnostics for Underserved Population (RADx-UP) to increase COVID-19 testing resources available to residents of US/Mexico border communities [19]. Simultaneously, in May of 2021, vaccine availability was increasing, the FDA had expanded the emergency use authorization for the Pfizer COVID-19 vaccine to include 12–15-year-olds, and by January of 2022 the United States Government would also make free at-home rapid antigen COVID-19 tests accessible (4). The key objective of this project was to understand practices, barriers, and facilitators to access and uptake of COVID-19 testing in these populations and partner with the community to develop and implement a culturally appropriate COVID-19 testing program. To achieve these objectives the CO-CREATE project engaged multiple community partners in completing a needs assessment to identify facilitators and barriers associated with developing a co-created COVID-19 testing site. From this needs assessment, participants who indicated being unable to test reported (1) being unable to get an appointment, (2) being worried about cost, and (3) being worried about being infected when going to a testing site [20].

Key to COVID-19 testing uptake within Latino/a communities is building trust between community members and those providing testing, highlighting the importance of (1) protecting participant personal information and informing individuals of how the information will be used, (2) providing legal protection at testing sites for undocumented immigrants, (3) having trusted networks for providing testing, such as faith-based organizations or community non-profits, and (4) offering culturally tailored preventative methods [20–23]. Accessible and effective testing programs involve the reduction or removal of testing barriers and the implementation of community-based and community-driven interventions [8].

CO-CREATE was designed as a community-driven and culturally-tailored COVID-19 testing model, with the ability to be adapted, and to adjust in response to the changing dynamics of the COVID-19 pandemic. While health and testing disparities experienced by Latino/a populations during the COVID-19 pandemic have been well described, few studies have reported the utilization of community-led COVID-19 testing models among Latinos/as [11, 14, 15, 24]. The objective of this report is to describe both the testing patterns and behaviors of study participants and the overall testing utilization of the CO-CREATE project within the Latino/a community.

## Methods

### Testing and sequencing procedure

To reduce testing barriers and facilitate access to testing, all testing staff were bilingual (English/Spanish) and/or were residents of the San Ysidro community, and all participant-facing materials were provided in English and Spanish. The testing site provided a culturally appropriate testing approach by physically being on the premises of a community-trusted FQHC, engaging community leaders as part of the CSAB, and ensuring staff understood and/or shared cultural norms with research participants. In partnership with a Federally Qualified Health Center [25] and a community-based social change organization [26] the CO-CREATE project established a 22- member Community and Scientific Advisory Board (CSAB) [23, 27]. The CSAB consisted of community organizers, promotores de salud (community health workers), clinic providers and administrators, and public health researchers who actively participated in a seven-session Theory of Change process, to identify the necessary conditions that must exist to eliminate COVID-19 testing disparities and specific actions to create those conditions, followed by a series of appreciative inquiry sessions to further advise program implementation [20, 23]. Although testing hours and availability evolved over the course of the study, walk-up no-cost COVID-19 polymerase chain reaction (PCR) testing was offered daily during usual clinic hours at booths located near the entrance of the partnering Federally Qualified Health Center. Testing was provided on a first-come first-served basis, individuals did not need to be affiliated with the clinic to be tested, and there were no age restrictions for testing. Marketing of the testing site location and hours was promoted via social media (i.e., Twitter (X) and Instagram), billboards located around the community, word of mouth (i.e., participants sharing information with friends and family), and flyers (i.e., flyers were placed around the clinic and local businesses). Testing guidance was provided to individuals based on symptom screening and previous test results according to CDC guidelines. Testing implementation was adapted overtime to ensure effectiveness of the program and responsiveness to CSAB and participant identified community needs. Adaptations were tracked throughout the project, including during the pre-implementation, early-and mid/late-implementation, and sustainment of the program.

Minimal demographic data required for reporting to the County of San Diego Health and Human Services Agency (i.e., name, date of birth, address, and phone number) and preferred method of contact were collected prior to testing. To ensure data accuracy, identifying information was verified by requiring participants to show proof of identification (e.g., identification card, driver’s license, or Mexican identification card). However, if proof of identification was unavailable, individuals were still able to test using self-reported identification information. Individuals undergoing testing were instructed by study staff on how to self-collect a nasal swab.

Collected swabs were sent to the UCSD EXCITE clinical laboratory for PCR testing for COVID-19 (EUA202755 and EUA210524). Samples underwent additional sequencing if they had a median Cq value < 30 for all three viral qPCR assay targets. Samples with a median Cq value > 30 had a notably higher failure rate on the sequencing pipeline and were not sequenced. In 384w plate format, 1.33uL of purified nucleic acid extract was used for amplicon sequencing with the IDT xGen (formerly SWIFT) SARS-CoV-2 library preparation kit (Integrated DNA Technologies, Coralville, IA, PNs: 10009832 Panel, 10009827 Core reaction enzymes). Superscript IV VILO (Thermo Scientific, Carlsbad, CA PN 11756500) was used in a 1:12 scaled down reaction. The IDT xGen kit was scaled down 1:6. Up to 1536 samples were pooled together, each with its own unique dual index (UDI) (IDT DNA PNs:10009796, 10009797, 10009798, & 10009799). The Normalase component of the library preparation was omitted. Regents were dispensed with a Dragonfly liquid handler (SPT Labtech, Melbourn, UK) and samples and reactions were assembled using a Mosquito HV 16 channel liquid handler (SPT Labtech). A Blue Washer (BlueCatBio NH Inc., Lebanon, NH) was used to wash the 384w plates during the Ampure XP (Beckman Coulter, CA) bead cleanups in the library preparation protocol. 1uL of the final 29uL library was equally pooled and index sequenced on a MiSeq instrument (Illumina Inc., San Diego, CA) using a Nano flow cell with single-end 50 bp reads. Based on index read depth, libraries were balanced by volume using a Mosquito X1 liquid handler (SPT Labtech). The resultant pools were sequenced using a paired-end 150 bp read runs on NovaSeq 6000 (Illumina Inc.) using SP or S4 flow cells. Sequencing data was filtered for quality and analyzed for strain identification using the C-view pipeline [28].

Test results were returned to individuals via their preferred method of communication within 1–3 days of sample collection. If the result was positive, a clinical provider contacted the participant by phone to provide guidance on testing family members, isolation period recommendations, and seeking further clinical care, and to answer any testing questions. Spanish-speaking participants were contacted through an interpreter or by a Spanish-speaking healthcare provider.

## Study enrollment and data collection

Prior to sample collection, individuals were invited to enroll in the CO-CREATE study. If individuals elected to become part of the study, they were consented by study staff and asked to complete an enrollment questionnaire regarding their experience, knowledge, attitudes, and beliefs of COVID-19 testing and vaccination. After survey completion, study participants were compensated $20 USD. Community members of all ages were eligible to participate except for those who were unable to provide assent or consent (e.g., those with severe developmental delays or disabilities) or did not have a legal guardian who could consent on their behalf. Participants were eligible to return for testing twice per week but were required to obtain their previous results prior to returning to re-test. Return participants were asked to complete a return survey, which consisted of an abbreviated set of questions regarding their experience, knowledge, attitudes, and beliefs of COVID-19 testing and vaccination. Participants were compensated $10 USD for every return survey completed.

Surveys could be completed using a paper or electronic form through a link from the Research Electronic Data Capture (REDCap) system [29]. The enrollment survey contained required Common Data Elements (CDEs) developed as part of the RADx-UP program and included basic demographic questions and questions related to the participant’s experiences and beliefs related to COVID-19 testing and vaccination along with their knowledge of COVID-19 disease and testing accessibility [30].

## Data sources, management, and analysis

Frequencies were compared between categorical variables using Chi Squared Tests and distributions for continuous variables were compared using t-tests and analyses of variance (ANOVA). Data were aggregated by testing week and visualized using histograms and line graphs to observe changes in testing and positivity rates over time. Aggregated zip code county-reported COVID-19 testing, and positivity data were provided by the County of San Diego Health and Human Services Agency. The data in REDCap were accessed using REDCap Python API for analysis and alignment with NIH CDE vocabularies. Additional analyses were performed using STATA17 (StataCorp, College Station, TX).

## Results

### CO-CREATE testing in the San Ysidro community

**Fig. 1:**
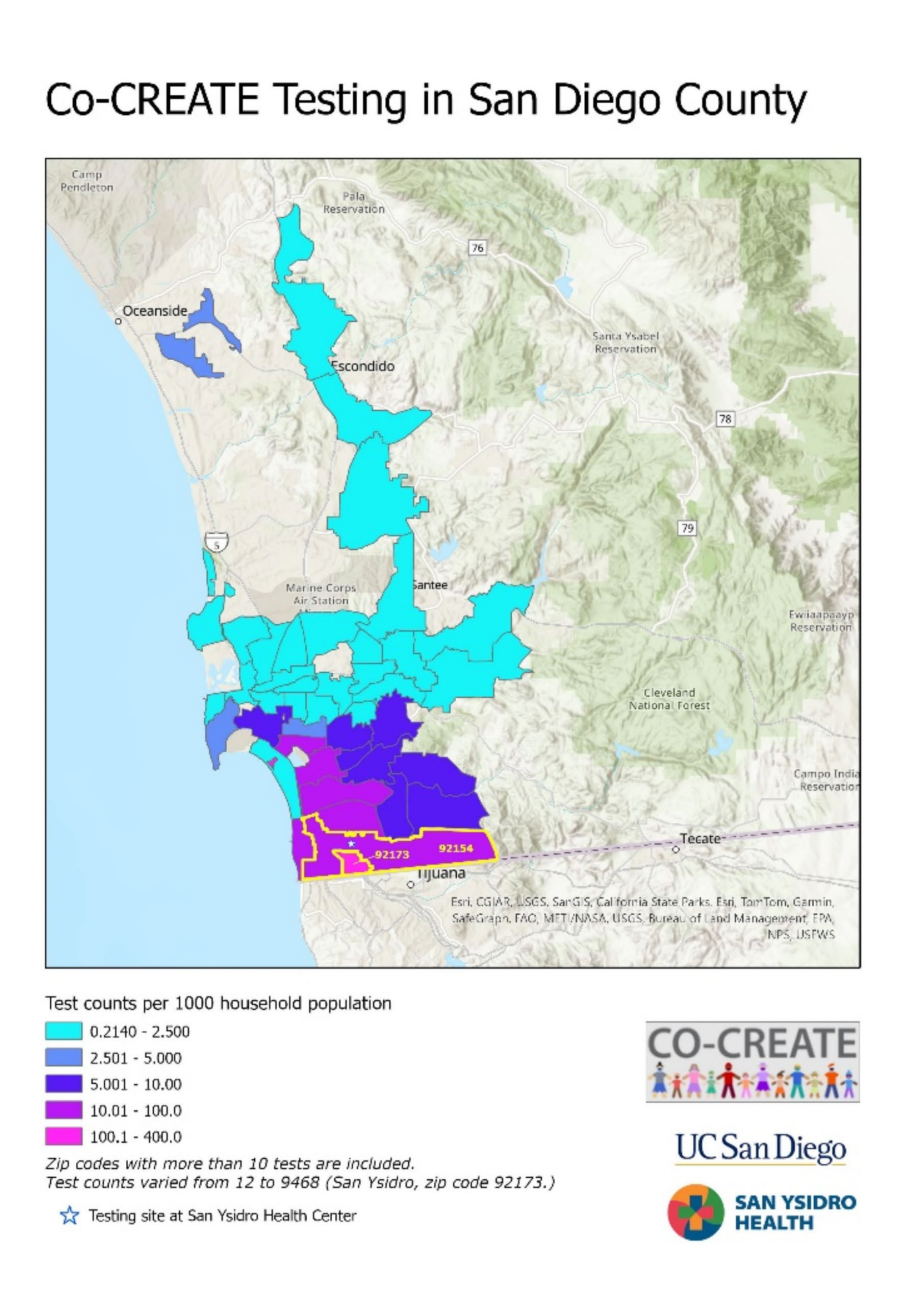
Testing density and reach. Count of COVID-19 test administered by CO-CREATE based on participants’ self-reported zip codes. Shades of purple and pink demonstrate higher concentration of testers residing in the southern region of San Diego County

Between May 1, 2021, and March 31, 2023, a total of 24,422 COVID-19 PCR tests were administered through the CO-CREATE project, of which 12.6% detected the SARS-Cov-2 virus and were classified as positive for COVID-19 infection (see Table 1). During the study period, individuals who identified 92,173 (San Ysidro) as their residential zip code accounted for 38.8% of all tests administered and 43.0% of all positive COVID-19 results (Table 1). Based on San Diego census data, nearly 17% of San Ysidro residents were tested for COVID-19 through CO-CREATE and approximately 12% enrolled in the CO-CREATE study and completed the survey (Fig. 1).

The proportion of COVID-19 PCR tests that detected SARS-CoV-2 virus was not significantly different between San Ysidro (14.0%) and San Ysidro-adjacent (12.9%) residents but was higher compared to non-San Ysidro (11.2%) residents (Table 1). The positivity rate among individuals who opted to complete the survey in addition to being tested was lower (10.7%) than those who opted for test only (19.2%) and did not complete the questionnaire (Table 1).

**Table 1:** COVID-19 PCR tests administered by zip code of residence

	92,713 Residents (San Ysidro)	92,154 Residents (San Ysidro-adjacent)	Non-San Ysidro Residents^1^	Total
Total Tests n	9,469	4,446	10,507	24,422
Positive tests, n (%)	1,322 (14.0)	573 (12.9)	1,181 (11.2)	3,076 (12.6)
Test & Survey participants	7,346	3,290	7,986	18,622
Positive tests, n (%)	838 (11.4)	351 (10.7)	776 (9.7)	1,965 (10.6)
Test only participants	2,123	1,156	2,521	5,800
Positive tests, n (%)	484 (22.8)	222 (19.2)	405 (16.0)	1,111 (19.2)

^1^including 490 unknown zip codes

**Fig. 2:**
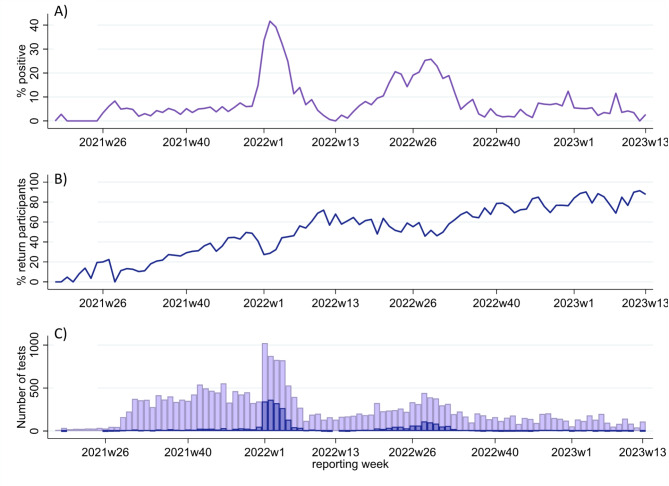
Testing details by reporting week. **(A)** Proportion of all tests administered that were COVID-19 positive, **(B)** Proportion of tests administered to individuals who were returning participants, and **(C)** Total tests (light purple) and total positive tests (blue) per reporting week

**Fig. 3:**
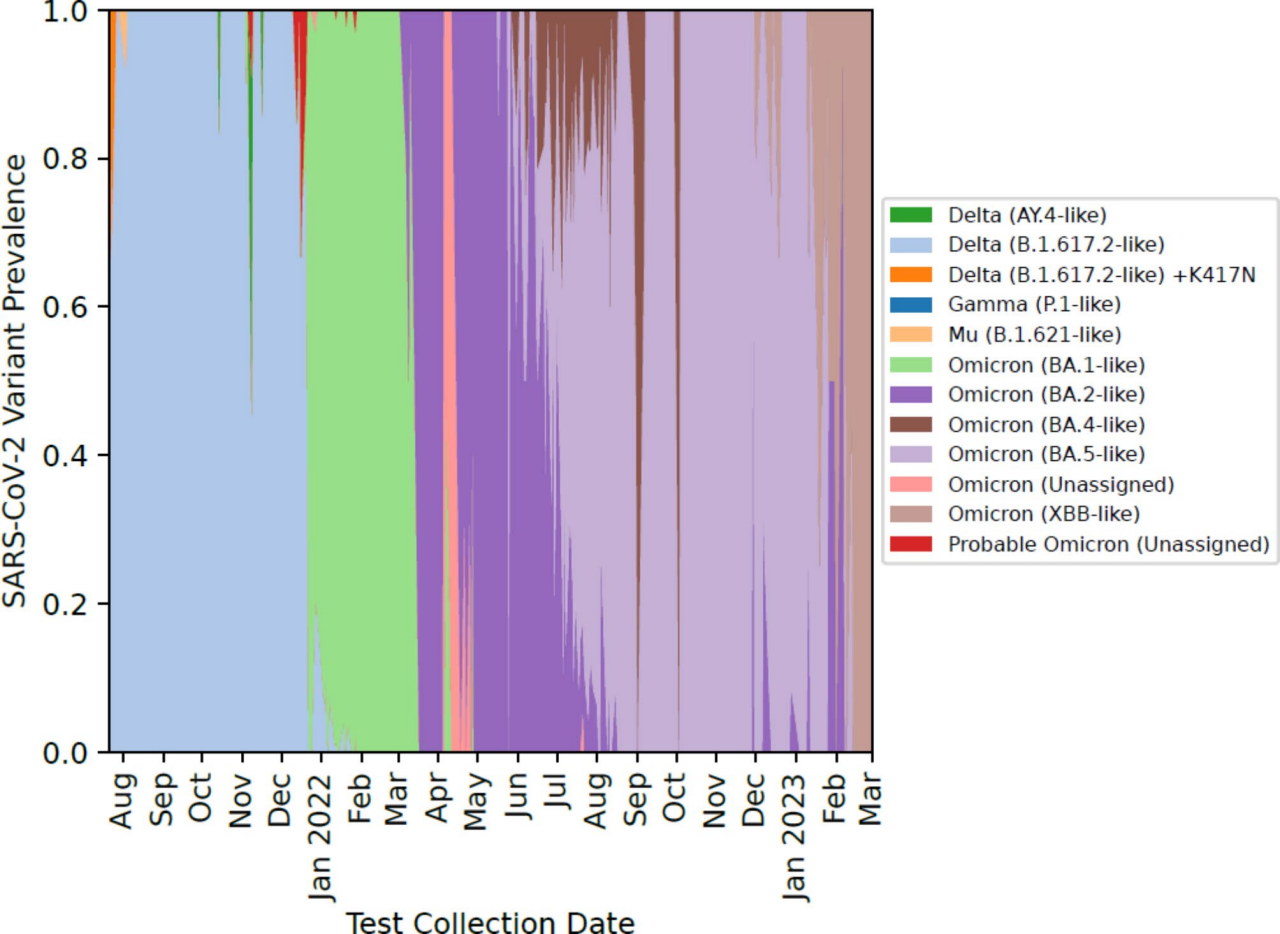
SARS-CoV-2 Variant prevalence by reporting month. COVID-19 variants identified from positive samples obtained from the CO-CREATE project

88% (2,732) of all positive samples were sequenced to identify COVID-19 variants. Positivity rates and testing demand varied over the course of the study and during the Omicron wave, in January 2022, weekly test positivity peaked above 40% (Fig. 2A and C). The proportion of COVID-19 PCR tests that detected the SARS-CoV-2 Delta B.1.617.2-like variant was higher between August 2022 to December 2022 (Fig. 3). From January 2022 to March 2023, various Omicron variants were more prevalent among CO-CREATE positive cases (Fig. 3). Overall, the proportion of return testers increased over the course of the study (Fig. 2B). However, the proportion of return testers dipped (and the proportion of first-time testers increased) during periods of high positivity, most notably during both the Omicron wave in January 2022 and again during the BA.5 wave in July 2022.

To evaluate project impact in context, we calculated the proportion of all COVID-19 tests administered by CO-CREATE to our target testing community, San Ysidro, out of all COVID-19 tests reported to the County of San Diego administered to San Ysidro residents. Using County data available for the 65 reporting weeks between May 1, 2021 and July 30, 2022 we determined that CO-CREATE accounted for 11.6% (7,703 of 66,497) of all tests and 12.6% (1,172 of 9,271) of positive results reported for San Ysidro residents during the analysis period. During the Omicron wave (1/2/22 − 2/12/22) CO-CREATE accounted for an even larger proportion of COVID-19 tests administered, 12.9% (1,911 of 14,780) of all tests and 14.5% (659 of 4,533) of all positive tests reported for San Ysidro residents were administered by CO-CREATE.

### Unique individual testing data

Of the 13,253 unique individuals tested, 26.9% (3,565 individuals) returned for at least one additional PCR test, resulting in 45.7% (11,169 of 24,422) of all PCR COVID-19 tests being administered to individuals who had returned for testing. Among unique individuals tested, 5,013 (37.8%) were San Ysidro residents and 2,580 (19.5%) were San Ysidro adjacent residents. Significantly more San Ysidro residents (24.5%) tested positive at least once for COVID-19 by PCR during the study period compared to San Ysidro-adjacent (21.1%) and non-San Ysidro residents (19.5%). Additionally, significantly more individuals from San Ysidro returned for testing (31.1%) compared to San Ysidro-adjacent residents (27.4%) and non-San Ysidro residents (23.0%). Approximately the same proportion of individuals identified as female among San Ysidro (59.9%), San Ysidro-adjacent (58.7%) and non-resident (56.8%) participants. The median age of San Ysidro residents was 34.5 years, 34.9 years for San Ysidro-adjacent residents, and 37.9 years among non-San Ysidro residents (Table 2).

**Table 2:** Unique Individuals

	92,713 Residents (San Ysidro)	92,154 Residents (San Ysidro-adjacent)	Non-San Ysidro Residents^1^	Total
Unique Individuals n	5,013	2,580	5,660	13,253
Positive at least once n (%)	1,226 (24.5)	544 (21.1)	1,105 (19.5)	2,875 (21.7)
Returned at least once n (%)	1,557 (31.1)	708 (27.4)	1,300 (23.0)	3,565 (26.9)
Returned > = 5 times n (%)	226 (4.5)	110 (4.2)	234 (4.1)	570 (4.3)
Female Sex n (%)	3005 (59.9)	1515 (58.7)	3214 (56.8)	7,734 (58.4)
Age median (IQR)	34.5 (15.6, 54.8)	34.9 (16.7, 54.0)	37.9 (20.9, 56.7)	35.9 (17.6, 55.6)

^1^including 248 unknown zip codes

**Fig. 4:**
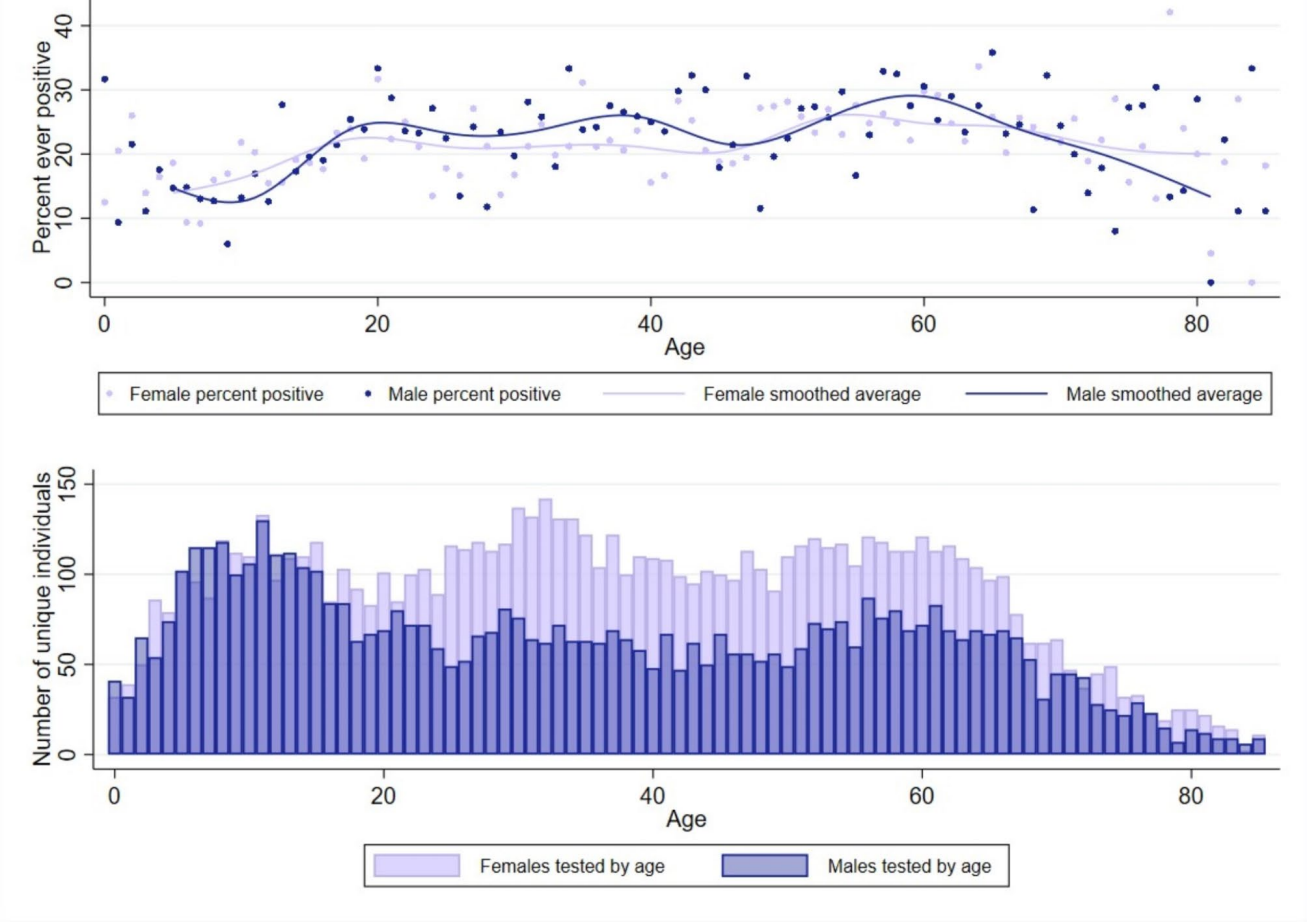
Ever positive and unique individuals by age and sex. **(A)** Percent of individuals who tested positive at least once during the study period. Each dot represents percent ever positive for each age year, by sex—light purple (female) and blue (male). Average ever positive over life course represented by median spline lines for each sex. **(B)** Bars represent total number of unique individuals that were tested by sex for each age year, light purple (female) and blue (male)

Evaluation of unique individuals revealed differences in testing by sex and age. Approximately 25% of all individuals tested were younger than 18 years of age. However, individuals younger than 18 years of age were less likely to ever test positive (16.6%) compared to adults (23.4%). Additionally, slightly less than half (49.4%) of individuals younger than 18 years of age were female, compared to adults where a majority (61.4%) of participants were female (Fig. 4B). However, the percent of participants who were ever positive during the study period was equal between males and females over the life span with the average female positivity rate (21.8%) comparable to the average male positivity rate (21.6%) (Fig. 4A).

### Survey participant testing behaviors and characteristics

Among all unique participants tested, 69% (9,147) individuals opted to complete a questionnaire exploring behaviors and beliefs surrounding COVID-19 disease and testing. 61% of survey respondents were female and 59% reported speaking Spanish at home (Table 3). Approximately half of survey respondents left responses blank regarding their household size, self-identified risk category, and primary reason for testing. However, among those who did respond, there were no statistically significant differences in these variables by zip code of residence. Being symptomatic (23.9%), having a known exposure (20.7%), and not having symptoms but wanting to know infection status (18.6%) were the most frequently identified reasons for testing.

**Table 3:** Survey participant characteristics

	92,713 Residents (San Ysidro)	92,154 Residents (San Ysidro-adjacent)	Non-San Ysidro Residents^1^	Total
Total Survey participants	3,506	1,748	3,893	9,147
Female sex	2,180 (62.2)	1,059 (60.6)	2,304 (59.2)	5,543 (60.6)
Spanish spoken in the home	2,073 (59.1)	1,009 (57.7)	2,135 (54.8)	5,217 (57.0)
Hispanic ethnicity	3,380 (96.3)	1,637 (93.5)	3,519 (90.0)	8,536 (93.1)
Age, median (IQR)	35.6 (15.3, 54.3)	36.2 (17.1, 52.6)	38.0 (21.4, 55.6)	36.7 (17.9, 54.5)
Household size				
Alone	203 (5.8)	92 (5.3)	319 (8.2)	614 (6.7)
With roommate or partner	253 (7.2)	135 (7.7)	328 (8.4)	716 (7.8)
Family with children	936 (26.7)	487 (27.9)	979 (25.1)	2,402 (26.3)
Family with 3 or 4 generations	211 (6.0)	132 (7.6)	223 (5.7)	566 (6.2)
Other/missing	1,903 (54.3)	902 (51.6)	2,044 (52.5)	4,849 (53.0)
Self-identified risk category				
Low risk	530 (15.1)	272 (15.6)	656 (16.9)	1,458 (15.9)
Medium risk	470 (13.4)	272 (15.6)	568 (14.6)	1,310 (14.3)
High risk	425 (12.1)	191 (10.9)	472 (12.1)	1,088 (11.9)
Unknown risk	512 (14.6)	227 (13.0)	513 (13.2)	1,252 (13.7)
Missing	1,569 (44.8)	786 (45.0)	1,684 (43.3)	4,039 (44.2)
Reasons for testing				
Required for work	197 (5.6)	89 (5.1)	253 (6.5)	539 (5.9)
Symptomatic	448 (12.8)	248 (14.2)	544 (14.0)	1,240 (13.6)
Exposure	401 (11.4)	224 (12.8)	446 (11.5)	1,071 (11.7)
Asymptomatic but wanted to know status	413 (11.8)	161 (9.2)	388 (10.0)	962 (10.5)
Other	239 (6.8)	117 (6.7)	298 (7.7)	654 (7.1)
Missing	1,808 (51.6)	909 (52.0)	1,964 (50.4)	4,681 (51.2)

^1^including 92 unknown zip codes

## Discussion

Several notable findings emerged from our analysis. Over the course of the study, nearly 17% of San Ysidro residents were tested for COVID-19 through the CO-CREATE project and over a quarter (26.9%) of individuals who tested with CO-CREATE returned for at least one additional PCR test. Additionally, testing conducted through CO-CREATE accounted for 11.6% of all tests administered and 12.9% of positives reported during the Omicron wave (as identified through sequencing data) to the County of San Diego among San Ysidro residents during the analysis period. These findings suggest that by the end of the study period, CO-CREATE was widely recognized and trusted by the local community as an accessible COVID-19 testing site. By ensuring the testing site location was near the entrance of a trusted Federally Qualified Health Center, community members were more likely to link CO-CREATE with a trusted healthcare provider. San Ysidro residents were more likely to receive a COVID-19 positive test result compared to non-San Ysidro residents, further underscoring that CO-CREATE provided a needed service to a community with a COVID-19 test-positive rate higher than that in the rest of the San Diego region. Finally, among adult test participants, more females tested with CO-CREATE (60.6%) compared to males, although the percentage of positive cases were equal between sexes (females 21.8% and males 21.6%),, aligning with previous findings suggesting women were more likely to access COVID-19 testing [24]. This was also likely influenced by the CO-CREATE testing site co-location with a FQHC with maternal and children health services.

These findings indicate a community-led project with accessible resources such as appropriate language material and personnel, could be successful in providing accessible COVID-19 testing to a Latino/a community during the COVID-19 pandemic. Latinos/as have been historically affected by systemic racism, shaping their social determinants of health, which was persistent throughout the COVID-19 pandemic [22, 31, 32]. As found by previous studies, providing a community-tailored approach to COVID-19 testing reduced barriers typically faced by the Latino/a community, including mistrust of testing procedures, non-permanent or transient testing locations, and reduced access to testing sites due to their inaccessibility by public transportation [4, 14, 21, 33]. CO-CREATE confirmed the importance of focusing on and adapting to community-identified needs [3, 20, 34, 35], to ensure equitable resource distribution and maintain sustainable testing resources within the San Ysidro community [25, 33].

### Implications and recommendations

Addressing testing barriers (i.e. hours of operation, trust in testing site location/operations, language barriers, etc.) in the Latino/a community, is key to designing and implementing sustainable testing. We recommend a focused approach to equitable access for both males and females, particularly adults between 18 and 66 years of age. Although this study did not identify reasons for why males were less likely to test, possible barriers to males testing could be related to work schedules interfering with testing site hours, personal perception of COVID-19 risk, or fear of losing work hours due to a positive test result [21]. It is vital to address barriers and implement facilitators to testing accessibility in Latino/a communities as. marginalized communities are dis-proportionately affected by the lack of or inadequate health information which can contribute to fear and mistrust, and ultimately impact an individual’s decision to seek care [6, 11, 15, 21]. CO-CREATE demonstrated successful utilization of a community-led COVID-19 testing by focusing on relationship-building with the community [35]. Like other studies, we found patients in Latino/a populations learn about COVID-19 testing sites through family and friend referrals [21]. Providing a space for community advocates through our CSAB meetings, such as community health workers or FQHC clinical providers allowed research and study staff to address testing disparities identified by the community, such as accessibility concerns rapidly [8, 21, 23, 24, 26]. Future studies should continue to focus on reducing health care testing disparities and work closely with communities to inform strategic selection of appropriate interventions to reduce these disparities.

## Limitations

Data collected for this manuscript were gathered from our project’s testing and positivity data and by administrating a survey to participants who consented into the study. The limitation of this study was not evaluating a control site to determine specific motivators for testing with CO-CREATE, however due to the project’s community-led design and rapid implementation this approach was not possible. In addition, we observed a higher test-positive rate in subjects who declined the survey and we speculate that persons who felt ill were less inclined to perform the survey as consent and completion of the survey was relatively long (30 min) which may have resulted in some reporting or information bias. Despite these limitations, CO-CREATE demonstrated the potential for successful testing models within the Latino/a community.

## Conclusion

Our study underscores the value of a community-vetted and co-designed testing program and is a replicable model for effectively responding to future pandemics and other public health crises. Although our main population consisted of Latinos/as this does not imply that the testing barriers and behaviors described in this study applies to all Latino/a communities. Furthermore, while our study population included equal numbers of male and female children, there were markedly more female than male adult participants and although not a novel finding, this highlights the importance of developing and implementing strategies to reduce sex-based disparities.

## Data Availability

No datasets were generated or analysed during the current study.

## References

[1] CDC Museum COVID-19 Timeline. [https://www.cdc.gov/museum/timeline/covid19.html]

[2] Graham G. <ArticleTitle Language=“En”>Addressing the disproportionate impact of COVID-19 on communities of Color. J Racial Ethn Health Disparities. 2021;8(2):280–2. 33742351 10.1007/s40615-021-00989-7 PMC7978471

[3] Chugg B, Lu L, Ouyang D, Anderson B, Ha R, D’Agostino A, Sujeer A, Rudman SL, Garcia A, Ho DE. Evaluation of Allocation Schemes of COVID-19 Testing Resources in a Community-Based Door-to-Door Testing Program. JAMA Health Forum. 2021;2(8):e212260. 35977196 10.1001/jamahealthforum.2021.2260 PMC8796878

[4] 2017. Demographic Profiles San Diego County [https://www.sandiegocounty.gov/hhsa/programs/phs/community_health_statistics/]

[5] Holden TM, Simon MA, Arnold DT, Halloway V, Gerardin J. Structural racism and COVID-19 response: higher risk of exposure drives disparate COVID-19 deaths among Black and Hispanic/Latinx residents of Illinois, USA. BMC Public Health. 2022;22(1):312. 35168585 10.1186/s12889-022-12698-9 PMC8845334

[6] Webb Hooper M, Nápoles AM, Pérez-Stable EJ. COVID-19 and Racial/Ethnic Disparities. JAMA. 2020;323(24):2466–7. 32391864 10.1001/jama.2020.8598 PMC9310097

[7] Khanna N, Klyushnenkova EN, Kaysin A. Association of COVID-19 With Race and Socioeconomic Factors in Family Medicine. J Am Board Fam Med. 2021;34(Suppl):S40–7. 33622817 10.3122/jabfm.2021.S1.200338

[8] Barry K, McCarthy M, Melikian G, Almeida-Monroe V, Leonard M, De Groot AS. Responding to COVID-19 in an Uninsured Hispanic/Latino Community: Testing, Education and Telehealth at a Free Clinic in Providence. R I Med J (2013) 2020, 103(9):41–46. 33126788

[9] Dalva-Baird NP, Alobuia WM, Bendavid E, Bhattacharya J. Racial and ethnic inequities in the early distribution of U.S. COVID-19 testing sites and mortality. Eur J Clin Invest. 2021;51(11):e13669. 34390487 10.1111/eci.13669 PMC8420583

[10] Galletly CL, Lechuga J, Dickson-Gomez JB, Glasman LR, McAuliffe TL, Espinoza-Madrigal I. Assessment of COVID-19-Related Immigration Concerns Among Latinx Immigrants in the US. JAMA Netw Open. 2021;4(7):e2117049. 34279648 10.1001/jamanetworkopen.2021.17049 PMC8290301

[11] Jacobson M, Chang TY, Shah M, Pramanik R, Shah SB. Racial and Ethnic Disparities in SARS-CoV-2 Testing and COVID-19 Outcomes in a Medicaid Managed Care Cohort. Am J Prev Med. 2021;61(5):644–51. 34412946 10.1016/j.amepre.2021.05.015 PMC8282435

[12] Mackey K, Ayers CK, Kondo KK, Saha S, Advani SM, Young S, Spencer H, Rusek M, Anderson J, Veazie S, et al. Racial and Ethnic Disparities in COVID-19-Related Infections, Hospitalizations, and Deaths: A Systematic Review. Ann Intern Med. 2021;174(3):362–73. 33253040 10.7326/M20-6306 PMC7772883

[13] COVID-19. Racial Disparities in Testing, Infection, Hospitalization, and Death: Analysis of Epic Patient Data [https://www.kff.org/coronavirus-covid-19/issue-brief/covid-19-racial-disparities-testing-infection-hospitalization-death-analysis-epic-patient-data]

[14] Grigsby-Toussaint DS, Shin JC, Jones A. Disparities in the distribution of COVID-19 testing sites in black and Latino areas in new York City. Prev Med. 2021;147:106463. 33647352 10.1016/j.ypmed.2021.106463 PMC9753026

[15] Hu T, Yue H, Wang C, She B, Ye X, Liu R, Zhu X, Guan WW, Bao S. Racial Segregation, Testing Site Access, and COVID-19 Incidence Rate in Massachusetts, USA. Int J Environ Res Public Health 2020, 17(24). 10.3390/ijerph17249528 PMC7766428 33352650

[16] Covid-19 cases by geography of residence. [https://www.sandiegocounty.gov/content/sdc/hhsa/programs/phs/community_epidemiology/dc/2019-nCoV/status/COVID19_Cases_by_Geography_of_Residence.html]

[17] Meneses Gutiérrez MM. Mexico-US local transborder micro-business methods resisting border travel restrictions in 2020. Lat Stud. 2021;19(3):400–4. 34121932 10.1057/s41276-021-00330-7 PMC8185495

[18] [https://www.gsa.gov/about-us/gsa-regions/region-9-pacific-rim/land-ports-of-entry/san-ysidro-land-port-of-entry]

[19] Williams NJ, Gill E, Punter MA, Reiss J, Goodman M, Shelley D, Thorpe LE. Rapid Community Engagement in Response to SARS-CoV-2 Funding Opportunities: New York City, 2020–2021. Am J Public Health. 2022;112(S9):S904–8. 36446061 10.2105/AJPH.2022.307072 PMC9707719

[20] Salgin L, Ayers LO, Burola M-L, Engler A-M, Osuna A, Gay L, Cain K, Stadnick N, Rabin B, Zaslavsky I et al. Perceived COVID-19 risk and testing experiences in the San Ysidro U.S./Mexico border region. Translational Behav Med 2023:ibac120. 10.1093/tbm/ibac120 PMC10314726 36999822

[21] Garcini LM, Pham TT, Ambriz AM, Lill S, Tsevat J. COVID-19 diagnostic testing among underserved Latino communities: Barriers and facilitators. Health Soc Care Community. 2022;30(5):e1907–16. 34719072 10.1111/hsc.13621 PMC8652902

[22] LeBrón AMW, Viruell-Fuentes EA. 21. Racism and the Health of Latina/Latino Communities. Racism: Science & Tools for the Public Health Professional. edn.: American Public Health Association; 2019.

[23] Stadnick NA, Cain KL, Oswald W, Watson P, Ibarra M, Lagoc R, Ayers LO, Salgin L, Broyles SL, Laurent LC, et al. Co-creating a Theory of Change to advance COVID-19 testing and vaccine uptake in underserved communities. Health Serv Res. 2022;57(Suppl 1):149–57. 35243622 10.1111/1475-6773.13910 PMC9108217

[24] Danielsen AC, Lee KM, Boulicault M, Rushovich T, Gompers A, Tarrant A, Reiches M, Shattuck-Heidorn H, Miratrix LW, Richardson SS. Sex disparities in COVID-19 outcomes in the United States: Quantifying and contextualizing variation. Soc Sci Med. 2022;294:114716. 35042136 10.1016/j.socscimed.2022.114716 PMC8743486

[25] About, Us. [https://www.syhealth.org/about-us]

[26] Rabin BA, Cain KL, Salgin L, Watson PL, Oswald W, Kaiser BN, Ayers L, Yi C, Alegre A, Ni J, et al. Using ethnographic approaches to document, evaluate, and facilitate virtual community-engaged implementation research. BMC public health vol. 2023;23:409. 10.1186/s12889-023-15299-2 PMC9974043 36855118

[27] The Global Action Research Center. [https://www.theglobalactionresearchcenter.org/]

[28] Karthikeyan S, Levy JI, De Hoff P, Humphrey G, Birmingham A, Jepsen K, Farmer S, Tubb HM, Valles T, Tribelhorn CE, et al. Wastewater sequencing reveals early cryptic SARS-CoV-2 variant transmission. Nature. 2022;609(7925):101–8. 35798029 10.1038/s41586-022-05049-6 PMC9433318

[29] Harris PA, Taylor R, Thielke R, Payne J, Gonzalez N, Conde JG. Research electronic data capture (REDCap)--a metadata-driven methodology and workflow process for providing translational research informatics support. J Biomed Inf. 2009;42(2):377–81. 10.1016/j.jbi.2008.08.010 PMC2700030 18929686

[30] Lomeli A, Escoto AA, Reyes B, Burola MLM, Tinoco-Calvillo S, Villegas I, Cohen AS, Laurent LC, Salgin L, Stadnick NA et al. Factors associated with COVID-19 vaccine uptake in a US/Mexico border community: demographics, previous influenza vaccination, and trusted sources of health information. Front Public Health 2023, 11. 10.3389/fpubh.2023.1163617 PMC10415906 37575117

[31] Moore KS. The Impact of COVID-19 on the Latinx Population: A Scoping Literature Review. Public Health Nurs. 2021;38(5):789–800. 33876506 10.1111/phn.12912 PMC8251024

[32] Nanchal R, Patel D, Guddati AK, Sakhuja A, Meersman M, Dalton D, Kumar G. Outcomes of Covid 19 patients-Are Hispanics at greater risk? J Med Virol. 2022;94(3):945–50. 34633096 10.1002/jmv.27384 PMC8662298

[33] Buro AW, Roman Candelaria K, Bailey R, Luna F, Albizu-Jacob A, Stern M, Redwine L. Exploration of Multilevel Barriers and Strategies That Affected Early COVID-19 Vaccination and Testing in Rural Latino Communities in Southwest Florida. Int J Environ Res Public Health 2022, 19(18). 10.3390/ijerph191811785 PMC9517188 36142059

[34] Oh DL, Meltzer D, Wang K, Canchola AJ, DeRouen MC, McDaniels-Davidson C, Gibbons J, Carvajal-Carmona L, Nodora JN, Hill L, et al. Neighborhood Factors Associated with COVID-19 Cases in California. J Racial Ethn Health Disparities. 2023;10(6):2653–62. 36376642 10.1007/s40615-022-01443-y PMC9662780

[35] Patel P J, Christofferson N, Goodlet KJ. Pharmacist-provided SARS-CoV-2 testing targeting a majority-Hispanic community during the early COVID-19 pandemic: Results of a patient perception survey. J Am Pharm Assoc (2003). 2022;62(1):187–93. 34465524 10.1016/j.japh.2021.08.015 PMC8373847

